# BCG-Based Vaccines Elicit Antigen-Specific Adaptive and Trained Immunity against SARS-CoV-2 and *Andes orthohantavirus*

**DOI:** 10.3390/vaccines10050721

**Published:** 2022-05-04

**Authors:** Jorge A. Soto, Fabián E. Díaz, Angello Retamal-Díaz, Nicolás M. S. Gálvez, Felipe Melo-González, Alejandro Piña-Iturbe, Mario A. Ramírez, Karen Bohmwald, Pablo A. González, Susan M. Bueno, Alexis M. Kalergis

**Affiliations:** 1 Millennium Institute of Immunology and Immunotherapy, Departamento de Genética Molecular y Microbiología, Facultad de Ciencias Biológicas, Pontificia Universidad Católica de Chile, Av. Portugal 49, Santiago 8320000, Chile; jasoto6@uc.cl (J.A.S.); fediaz4@uc.cl (F.E.D.); aretamal@bio.puc.cl (A.R.-D.); nrgalvez@uc.cl (N.M.S.G.); felipe.bqco@gmail.com (F.M.-G.); llpina@uc.cl (A.P.-I.); maramirez8@uc.cl (M.A.R.); kbohmwald@uc.cl (K.B.); pagonzalez@bio.puc.cl (P.A.G.); sbueno@bio.puc.cl (S.M.B.); 2 Millennium Institute on Immunology and Immunotherapy, Departamento de Ciencias Biológicas, Facultad de Ciencias de la Vida, Universidad Andrés Bello, Santiago 7550196, Chile; 3 Departamento de Endocrinología, Facultad de Medicina, Pontificia Universidad Católica de Chile, Santiago 8320000, Chile

**Keywords:** recombinant BCG, SARS-CoV-2, *Andes orthohantavirus*, immune response, trained immunity

## Abstract

**Background:***Mycobacterium bovis* Bacillus Calmette-Guérin (BCG) is a live attenuated vaccine mainly administered to newborns and used for over 100 years to prevent the disease caused by *Mycobacterium tuberculosis* (*M. tb*). This vaccine can induce immune response polarization towards a Th1 profile, which is desired for counteracting *M. tb*, other mycobacteria, and unrelated intracellular pathogens. The vaccine BCG has been used as a vector to express recombinant proteins and has been shown to protect against several diseases, particularly respiratory viruses. **Methods:** BCG was used to develop recombinant vaccines expressing either the Nucleoprotein from SARS-CoV-2 or *Andes orthohantavirus*. Mice were immunized with these vaccines with the aim of evaluating the safety and immunogenicity parameters. **Results:** Immunization with two doses of 1 × 10^8^ CFU or one dose of 1 × 10^5^ CFU of these BCGs was safe in mice. A statistically significant cellular immune response was induced by both formulations, characterized as the activation of CD4^+^ and CD8^+^ T cells. Stimulation with unrelated antigens resulted in increased expression of activation markers by T cells and secretion of IL-2 and IFN-γ, while increased secretion of IL-6 was found for both recombinant vaccines; all of these parameters related to a trained immunity profile. The humoral immune response elicited by both vaccines was modest, but further exposure to antigens could increase this response. **Conclusions:** The BCG vaccine is a promising platform for developing vaccines against different pathogens, inducing a marked antigen-specific immune response.

## 1. Introduction

*Mycobacterium bovis* Bacillus Calmette-Guérin (BCG) is an attenuated strain of *M. bovis* and the only available vaccine for preventing and treating the infection caused by *M. tuberculosis* [1,2,3]. This live-attenuated vaccine has been widely used since 1921, and it is one of the most used vaccines worldwide [4,5]. The vaccine BCG is commonly administered to newborns, and its safety and efficacy have been widely reported across multiple demographics [4,5]. Accordingly, this vaccine is currently administrated in over 200 countries [4,5]. The vaccine BCG can induce a marked Th1 polarization of T cells, characterized by the antigen-specific secretion of IFN-γ and an enhanced antiviral profile in both human and murine models [1,6]. In addition, the polarization of a Th1 profile in CD4^+^ T cells makes this vaccine an excellent candidate for overcoming Th2-biased immune responses seen against some pathogens in neonates [7].

The vaccine BCG can also induce protection against unrelated pathogens, a capacity termed trained immunity, which supports its use in neonates [8]. Trained immunity is a non-specific immune response arising from vaccination or infection that enhances the responsiveness of immune cells such as monocytes, macrophages, and natural killer (NK) cells to further heterologous infections [9,10]. This phenomenon occurs in the hematopoietic stem cells of the bone marrow, and the effects can be seen in the myeloid progenitors [9,11,12]. The trained phenotype observed in immune and endothelial cells has been attributed to epigenetic modifications [9,11]. Among the known trained immunity inducer are β-glucans [12], *Candida albicans* [13], oxLDL (oxidized low-density lipoprotein) [14], and BCG [15], among others. In addition, BCG vaccination can modify the phenotype of monocytes that secrete a higher amount of cytokines such as TNF-α, IFN-γ, and IL-1β upon exposure to non-mycobacterial pathogens [15]. Monocytes and other immune cells activated upon BCG immunization exhibit increased histone H3 lysine 4 mono- and tri-methylation (H3K4me and H3K4me3, respectively) in the promoters of genes encoding for pro-inflammatory proteins such as *tnfα*, *il6*, and *tlr4* [9,15]. This methylation results in increased accessibility for the transcription machinery, therefore increasing the expression of these genes [9,15]. Immunization by BCG has been reported to modulate diseases caused by viral infections [16]. In a case-control study, BCG vaccination reduced the severity of acute lower respiratory tract infections caused by hRSV [17].

Severe Acute Respiratory Syndrome Coronavirus 2 (SARS-CoV-2) is the emerging pathogen responsible for the Coronavirus Disease 2019 (COVID-19) pandemic [18,19,20,21,22]. This virus was first described in Wuhan, China, in December 2019 [23]. SARS-CoV-2 infects the upper and lower respiratory tracts, and this disease is characterized by several systemic symptoms [23]. Among the most common symptoms fever, cough, fatigue, headache, sore throat, shortness of breath; although, Acute Respiratory Distress Syndrome (ARDS), acute cardiac injury, anaemia, predisposition to secondary infections, and central nervous system damage (CNS) are reported [23,24,25].

SARS-CoV-2 belongs to the *Coronaviridae* family, genus *Betacoronavirus*, and is an enveloped, single-stranded, non-segmented, positive-sensed RNA virus [22,26,27]. Its genome size is about 29.9 kb, and it encodes for 36 proteins with 26 non-structural proteins (NSP) obtained from ORF1a (11 NSP) and ORF1b (15 NSP); 4 structural proteins (Spike, S; Envelope, E; Membrane, M; and Nucleoprotein, N); and at least six accessory proteins [22,26,27]. Infections by SARS-CoV-2 are usually characterized by significant lymphopenia, with depletion of CD4^+^ and CD8^+^ T cells [26]. Remarkably, studies have shown that CD4^+^ T cells generated upon infection were mostly specific against the M, N, and S proteins of this virus, with comparable numbers, proliferation capacities, and proportions of these cells in several responding patients [26,27]. However, the production of IFN-γ, IFN-β, and TNF-α was reduced in specific anti-N-SARS-CoV-2 cells compared to other T cells [26,27].

On the other hand, viruses from the *Orthohantavirus* genus are mostly pathogens responsible for two relevant clinical diseases: haemorrhagic fever with renal syndrome (HFRS) in Europe and Asia and the Hantavirus Cardiopulmonary Syndrome (HCPS) in the Americas. The HCPS case fatality rates can reach 20–40% [28,29]. Several *Orthohantavirus* species have been recognized as aetiological agents of HCPS, including the *Sin Nombre orthohantavirus* (SNV) and *Andes orthohantavirus* (ANDV) as the most relevant species in North America and South America, respectively [30,31]. Clinically, HCPS is characterized by an initial short prodromal period, with flu-like symptoms such as fever, headache, cough, myalgia, and gastrointestinal discomfort [29,32]. The patient may quickly progress to a cardiopulmonary stage following the prodrome, with severe dyspnoea, pulmonary oedema, and cardiogenic shock [33]. Patients who survive the cardiopulmonary stage enter the convalescent phase and slowly recover pulmonary function [33].

*Orthohantavirus* belongs to the family *Hantaviridae*, and order *Bunyavirales*, and is a single-stranded RNA virus with a tri-segmented genome. The ANDV viral genome encodes four viral proteins: A large genome (L) segment encodes for RNA-dependent RNA polymerase, a medium (M) segment encodes for Gn and Gc glycoproteins, and a Small (S) segment encodes for a nucleoprotein (N) [34]. *Orthohantavirus* is transmitted to humans by inhaling aerosolized excreta from wild infected rodents, their natural reservoirs [35,36]. Nevertheless, person-to-person transmission is also described for ANDV [37,38]. Mononuclear infiltrates of high cytokine-producing cells are present in the lungs of HCPS-deceased patients [39]. Additionally, a cytokine storm response is described by different groups, with high levels of IL-6 in deceased individuals [40,41]. A marred and dysbalanced Th1/Th2 immune response towards the virus has been associated with AEC dysregulation in clinical studies and animal models of HCPS infection [42,43]. The severe immune dysregulation after ANDV infection likely contributes to capillary leakage and the consequent organic dysfunction reported in HCPS cases.

Here, we tested whether two new recombinant BCGs (rBCGs) expressing the N-SARS-CoV-2 protein (rBCG-N-SARS-CoV-2) or the N-ANDV protein (rBCG-N-ANDV) are safe and immunogenic in mice. Our results show that immunization with each recombinant BCG is safe and well-tolerated. Moreover, both vaccines induce an antigen-specific cellular response characterized by the activation of CD4^+^ and CD8^+^ T cells and the secretion of specific antibodies against the N-proteins of both viruses. Finally, immunization with either non-recombinant or recombinant BCG resulted in an unspecific enhanced immune response, as evidenced by increased secretion of pro-inflammatory cytokines and T cells activation upon stimulation with an unrelated antigen.

## 2. Materials and Methods

### 2.1. Generation of Recombinant Mycobacterium bovis BCG Strains Expressing the Nucleoprotein of SARS-CoV-2 or ANDV

To generate an rBCG expressing the Nucleoprotein of SARS-CoV-2 (N-SARS-CoV-2), the sequence of this gene was synthesized by GenScript. Then, the DNA coding sequence of the N-SARS-CoV-2 protein was cloned in the pMV361 plasmid through restriction enzymes. To generate an rBCG strain expressing the Nucleoprotein of ANDV (N-ANDV), we defined an in-silico cloning strategy. The *n-andv* gene was obtained from an ANDV sequence from the CHI-7913 isolate and cloned into a pMV361 using the Gibson Assembly Cloning Kit (New England Biolabs #E5510, MA, USA). The presence of the genes in both the pMV361-N-SARS-CoV-2 and pMV361-N-ANDV constructions was confirmed by PCR and Sanger sequencing. Finally, the pMV361-N-SARS-CoV-2 and pMV361-N-ANDV plasmids were used to transform a wild-type BCG strain Danish 1331 (BCG-WT) through electroporation. Further details can be found in the Supplementary Material.

### 2.2. Expansion and Characterization of rBCG Strains

The rBCG-N-SARS-CoV-2 and the rBCG-N-ANDV colonies were grown on supplemented a 7H9 liquid medium (Sigma-Aldrich, M0178) for three weeks or until reaching an optical density (O.D. 600 nm) between 0.8 to 1. Final stocks were stored at 4 × 10^8^ CFU/vial or 4 × 10^5^ CFU/vial at 80 °C. Further details can be found on the Supplementary Material. The PCR and sequencing confirmation of both recombinant BCG strains were performed. The primers used to corroborate the presence of the genes by PCR can be found in the Supplementary Material.

### 2.3. Protein Expression Evaluation through Western Blot

We performed Western blot assays to characterize the expression of the N-SARS-CoV-2 and N-ANDV by the recombinant BCGs. Details on the protein extraction can be found on the Supplementary Material. A total of 20 µg of purified protein or total protein extracts were loaded into SDS-PAGE gels, according to what was reported previously [44,45,46,47], and run for 30 min at 90 V and then for 90 min at 120 V. Then SDS-PAGE was transferred into a nitrocellulose membrane using a Semi-Dry Power Blotter Transfer System (Thermo Fisher, MA, USA). Upon transfer, the membranes were blocked and then incubated with a mouse monoclonal anti-N antibody for SARS-CoV-2 diluted 1:500 (Sinobiological, cat 40143-MM05) or a monoclonal anti-N-ANDV mouse IgG1 antibody diluted 1:1000 (Austral Biologicals, cat HNM-6021DZ1-5). Both antibodies were incubated O.N at 4 °C. After washing a Goat anti-mouse-HRP antibody (Thermo Fisher, MA, USA) was incubated in a 1:2000 dilution at room temperature for one hour. The membranes were washed and a chemiluminescence reaction was performed with an ECL-Kit (Thermo Fisher, MA, USA). The membrane was visualized in a live view MyECL Imager (Thermo Fisher, MA, USA, cat 32106). Further details can be found in the Supplementary Material.

### 2.4. Mice, Immunization, and Safety Evaluation

**Figure 1:**
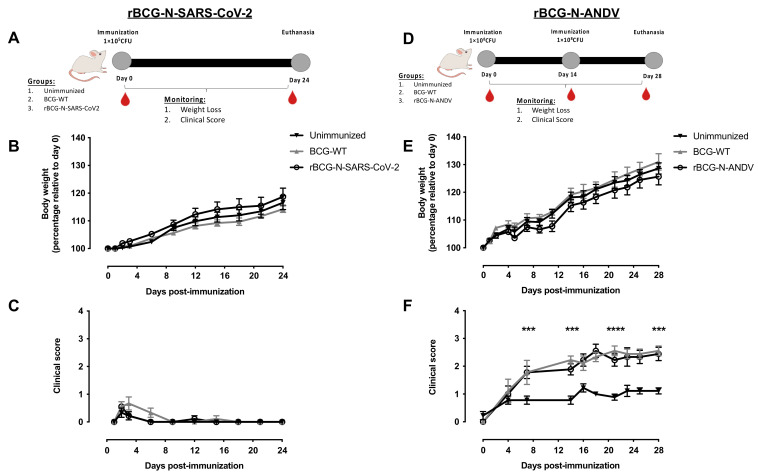
Determination of safety parameters after the administration of recombinant vaccines. The experimental schedule of immunization for rBCG-N-SARS-CoV-2 (**A**) and rBCG-N-ANDV (**D**) is presented. Safety parameters such as monitoring of weight loss for rBCG-N-SARS-CoV-2 (**B**) or rBCG-N-ANDV (**E**) and the clinical score for rBCG-N-SARS-CoV-2 (**C**), or rBCG-N-ANDV (**F**) are shown. Data are representative of 3 independent experiments. Each experiment considered a total of three mice per group. Statistical differences were assessed by one-way ANOVA with a *post hoc* Tukey test. Differences were only found in (**F**), with the BCG-WT and the rBCG-N-ANDV being statistically higher than the unimmunized mice. ***: *p* < 0001; ****: *p* < 00001.

Six- to eight-week-old BALB/cJ mice from the animal facility at Pontificia Universidad Católica de Chile were randomly distributed and harboured in pathogen-free conditions. In order to evaluate the safety and immunogenicity of the rBCG-N-SARS-CoV-2 strain, mice were immunized in a single dose immunization scheme, as was previously reported for other rBGCs [45,48]. Six- to eight-week-old BALB/cJ male mice were vaccinated by subcutaneous (sc) injection with 1 × 10^5^ CFU of rBCG-N-SARS-CoV-2, BCG-WT, or 1X PBS used as a vehicle in a final volume of 100 μL per dose. Twenty-four days after the first immunization, mice were anesthetized with ketamine and xylazine (80 mg/kg and 4 mg/kg, respectively) and euthanized. Blood samples were obtained from these animals before immunization (pre-immune), on day 14 post-immunization (14 dpi), and at the final time point (24 dpi) (Figure 1A). Spleens and lymph nodes were obtained after euthanasia. An experimental setup following the same conditions as the one described for the rBCG-N-SARS-CoV-2 but using a mix of two recombinant BCGs instead (one expressing the N-protein and another expressing the S-protein of SARS-CoV-2) was also evaluated.

To evaluate the safety and immunogenicity of the rBCG-N-ANDV, mice were immunized with two doses of 1 × 10^8^ CFU of BCG WT or rBCG-N-ANDV, or 1X PBS as a vehicle in a final volume of 100 μL per dose, with each dose separated by two weeks, as was previously used for other rBGCs [46,49,50,51]. This schedule differs from the one used for the rBCG-N-SARS-CoV-2 vaccine because ANDV causes a more severe infection than SARS-CoV-2, and therefore we started with a higher dose. Twenty-eight days after the first immunization, mice were anesthetized with ketamine and xylazine (80 mg/kg and 4 mg/kg, respectively) and euthanized. Blood samples were obtained from these animals before immunization (pre-immune), at day 14 pre-booster (14 dpi), and at the final time point (28 dpi) (Figure 1D). Spleens were obtained after euthanasia for both immunization schemes and used for cellular analyses. For both vaccines evaluated, safety parameters such as weight loss and clinical scores of the mice were monitored daily.

### 2.5. Bone Marrow-Derived Dendritic Cell (BMDCs) Cultures

Dendritic cells (DCs) were differentiated from bone marrow precursors and then used as antigen-presenting cells for T cells ex vivo recall stimulation, as reported before [52,53]. Further information can be found on the Supplementary Material. The DC differentiation was routinely assessed by flow cytometry.

### 2.6. T Cell Purification

Spleens and lymph nodes from mice were obtained to purify total T cells, as also described before [48,50]. Briefly, these organs were homogenized through a 70 μm cell strainer. Then, erythrocytes were lysed with ACK for 5 min at RT and centrifuged for 5 min at 400× *g* at 4 °C. Next, cells were resuspended in RPMI 1640 medium and counted. A total of 3 × 10^7^ cells were resuspended in 1XPBS, 2 mM EDTA, and 0.5% BSA (PEB buffer). T cells were purified from these mixtures of cells using a Pan T cell MACS kit (Miltenyi Biotech, Bergisch Gladbach, Germany), following the manufacturer’s instructions.

### 2.7. Co-Culture Stimulation Assay

A total of 1 × 10^5^ DCs/well were plated in 96-well plates. These cells were pulsed with the different stimuli used in these assays. The stimulus considered the N-SARS-CoV-2 purified protein in two different concentrations (10 µg/mL and 20 µg/mL—Genscript Biotech, NJ, USA); total protein extract from BCG-WT strain or PPD (10 µg/mL); total protein extract from rBCG-N strain (10 µg/mL); purified LPS (0.5 ng/mL—a concentration equivalent to the one detected in the purified recombinant proteins used in the assay); Concanavalin A (3 µg/mL); and anti-CD3/CD28 (1 mg/mL and 5 mg/mL, respectively). After 2 h of incubation, 2 × 10^5^ purified T cells were added to the DCs (T cells/DCs ratio equal to 2:1), as was previously described [54,55]. We also tested T cells/DCs ratios equal to 1:2 and 1:1, where we did not detect a significant T cell activation (data not shown). These cells were co-cultured for 72 h at 37 °C, with 5% CO_2_. After incubation, plates were centrifuged and then recovered for flow cytometry assays, and supernatants were stored at −80 °C for ELISA assays.

### 2.8. Ex Vivo T Cell Stimulation

Spleens were homogenized in RPMI medium, red blood cells were lysed, and cells were resuspended in RPMI medium. Then, an ex vivo antigen recall assay was performed on splenocytes. Briefly, 5 × 10^5^ cells were plated in a 96-well plate with complete RPMI and incubated with N-ANDV protein [10 μg/mL], PPD [20 μg/mL], and Concanavalin A (3 μg/mL) as a positive control. After 72 h, plates were centrifuged for 5 min at 300× *g* at 4 °C, and supernatants were collected and stored.

### 2.9. Flow Cytometry Evaluation

To characterize the activation of CD4^+^ and CD8^+^ T cells from the co-culture assays or the splenocytes cultures, cells were centrifugated for 5 min at 400× *g* at 4 °C. Then, samples were stained to evaluate lymphoid populations, as indicated in the Supplementary Material. Samples were acquired in a BD LSR-Fortessa-X20 (BD Biosciences, NJ, USA) and analyzed using FlowJo v10.6.2 software (BD Biosciences, NJ, USA).

### 2.10. Quantification of Cytokines Secreted during Co-Cultures by ELISA

To quantify the cytokines secreted after the co-culture of T cells and DCs, supernatants were collected and used directly (undiluted) to detect IL-2 (BD Biosciences #555148, NJ, USA), IL-4 (BD Biosciences #555232, NJ, USA), and IFN-γ (BD Biosciences #555138, NJ, USA) following the instructions of the manufacturer. Plates were revealed using 1 mg/mL of 3,3′,5,5′-tetramethylbenzidine (TMB, BD Biosciences) at RT, protected from the light, for 15 min. Then, the reaction was stopped by adding 50 µL of 2 N H_2_SO_4_. Plates were analyzed in an ELISA reader at 450 nm and 560 nm (Multiskan Ex, Thermo Fisher, MA, USA).

### 2.11. Quantification of Specific IgG against SARS-CoV-2 and ANDV Antigens by ELISA

To quantify the antigen-specific circulating IgG antibodies in immunized mice, 96 well ELISA plates were performed as previously reported [46,56] and with adaptations indicated in the Supplementary Material. Plates were analyzed in an ELISA reader at 450 nm and 560 nm (Multiskan Ex, Thermo Fisher, MA, USA).

### 2.12. Statistical Analyses

Statistical differences were assessed through one-way or two-way ANOVAs followed by Tukey post-hoc tests. Differences between means were statistically significant when the calculated *p*-value was <0.05. Data are presented as mean ± standard error of the mean (SEM) to represent the accuracy of the estimation of our sample means to the actual population mean. The significance of each statistic test is shown in the legend of each figure. All statistical analyses were performed using GraphPad Prism 9.1.0 Software (Dotmatics, CA, USA).

## 3. Results

### 3.1. rBCG Strains Express Either the N-SARS-CoV-2 or N-ANDV Proteins

We developed vaccine prototypes based on recombinant BCGs that express either the N-SARS-CoV-2 or N-ANDV proteins. Reference sequences of nucleoprotein genes were cloned into the integrative *Mycobacterium* plasmid pMV361, allowing constitutive expression controlled by an HSP60 promoter [57]. Transforming colonies were selected upon transformation, and PCR screening was performed. Positive clones were confirmed by Sanger sequencing, and a candidate clone was chosen to perform further assays. After sequencing the clones obtained, we chose those with a correct open reading frames to evaluate protein expression through western blot. These evaluations confirmed that one clone was obtained for the rBCG-N-SARS-CoV-2 strain, while two clones were obtained for the rBCG-N-ANDV (Supplemental Figure S1). For the rBCG-N-ANDV, clone 2 was chosen to perform the immunization assays because it showed a higher concentration of the target protein, as suggested by the increase in intensity identified in the western blot compared to clone 1.

### 3.2. The Administration of rBCG-N-SARS-CoV-2 or rBCG-N-ANDV Is Safe and Well-Tolerated in Mice

Different experimental settings were performed to evaluate the safety and tolerance of these vaccines in mice. We evaluated immunization schedules with a single dose or a booster dose at 14 or 28 days after the first immunization, using either 1 × 10^5^ CFU or 1 × 10^8^ CFU of the rBCGs. For the rBCG-N-SARS-CoV-2, we chose to explore the immunization schedule further, considering a single immunization with 1 × 10^5^ CFU the rBCG-N-SARS-CoV-2, as this dose was well-tolerated and is homologous to that currently being administered to humans [58]. As controls for this immunization schedule, mice were administered with 1 × 10^5^ CFU of BCG-WT or 1X PBS (unimmunized). For this experimental setup, samples were harvested 24 days post-immunization (Figure 1A). On the other hand, for the rBCG-N-ANDV, we chose to explore the immunization schedule further, considering two doses of 1 × 10^8^ CFU of rBCG-N-ANDV, with each dose separated by two weeks. This is in line with previous reports evaluating rBCGs in pre-clinical models [45,46,48,49,50,51]. As controls for this immunization schedule, mice were administered two doses of 1 × 10^8^ CFU of BCG-WT or 1X PBS on the same days. Samples for this setup were harvested on day 28 post-immunization (Figure 1D).

To assess the safety of the vaccine formulations, animals were monitored according to a clinical score sheet considering systemic and local reactions to vaccination. Mice immunized with BCG-WT or rBCG-N-SARS-CoV-2 showed no difference in weight monitoring compared to unimmunized mice (Figure 1B). A similar response was observed in the mice immunized twice with 1 × 10^8^ CFU of BCG-WT or rBCG-N-ANDV (Figure 1E). In addition, no significant adverse reactions were observed in immunized animals throughout the study, neither with BCG-WT, rBCG-N-SARS-CoV-2, or the rBCG-N-ANDV vaccines (Figure 1C,F). Immunized and unimmunized groups did not display any behavior or physiological alterations and had similar clinical scores to those of unimmunized animals. These observations suggest that the rBCG-N-SARS-CoV-2 and rBCG-N-ANDV formulations are safe in the BALB/c mouse model.

### 3.3. Administration of One or Two Doses of rBCG Promotes the Activation of T Cells

**Figure 2:**
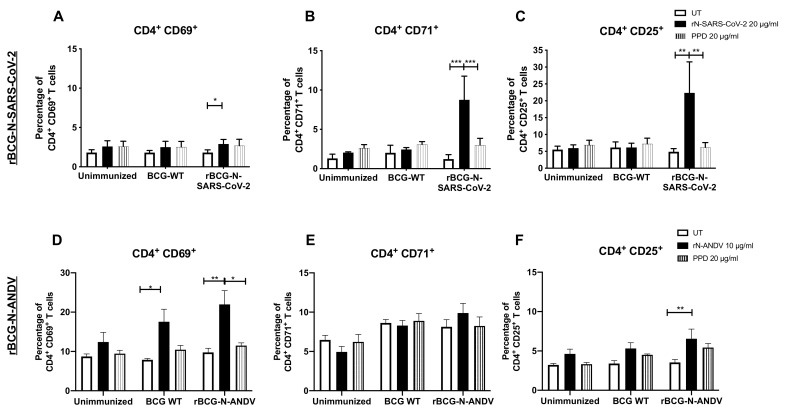
Determination of CD4^+^ T cells activation ex vivo after immunization. The determination of three different activation markers, CD69, CD71, and CD25, was performed ex vivo in mice immunized with each of the recombinant BCG vaccine candidates. For rBCG-N-SARS-CoV-2, the activation of CD69 (**A**), CD71 (**B**), and CD25 (**C**) were evaluated in CD4^+^ T cells. For rBCG-N-ANDV, the activation of CD69 (**D**), CD71 (**E**), and CD25 (**F**) were evaluated in CD4^+^ T cells. Data are representative of 3 independent experiments. Each experiment considered a total of three mice per group. Statistical differences were assessed by two-way ANOVA with a *post hoc* Tukey test. *: *p* < 0.05; **: *p* < 0.01; ***: *p* < 0.001.

To evaluate the induction of a specific T cell-mediated immunity, both CD4^+^ and CD8^+^ T cells were evaluated in ex vivo antigen stimulation assays with the corresponding recombinant N proteins. For rBCG-N-SARS-CoV-2, total T cells were purified from splenocytes and lymph nodes and co-cultured with dendritic cells for 72 h using the corresponding N antigens. A modest increase in the expression of the CD69 activation marker was detected in these cells by flow cytometry compared to CD71 and CD25. For CD69, statistically significant differences were found when the cells were stimulated with rN-SARS-CoV-2 compared to unstimulated cells (*p* = 0.0280) (Figure 2A). A three-fold increase was found for the expression of the CD71 activation marker when these cells were stimulated with the rN-SARS-CoV-2 protein, although no statistically significant differences were observed between the groups (Figure 2B). The highest activation level was reported for the CD25 marker, with about 20–30% of CD4^+^ T cells expressing this molecule. Statistically significant differences were found with the rN-SARS-CoV-2 stimulus compared to the untreated (*p* = 0.0004) and PPD-stimulated (*p* = 0.0059) T cells purified from rBCG-N-SARS-CoV-2-immunized mice (Figure 2C).

For rBCG-N-ANDV, total splenocytes were stimulated with the corresponding antigens for 72 h. When we evaluated the activation of T cells, a significant increase of CD4^+^CD69^+^ T cells was observed in the group immunized with rBCG-N-ANDV using the rN-ANDV protein stimulus compared with untreated (*p* = 0.0059) or PPD-stimulated cells (*p* = 0.0281) (Figure 2D). Interestingly, a significant increase was observed for the group immunized with BCG-WT using the rN-ANDV protein stimulus compared with untreated cells (*p* = 0.0390), suggesting an unspecific activation of T cells in line with a trained immunity profile. Similar expression levels of the CD71 marker were found in all the groups evaluated, without significant differences identified (Figure 2E). For the percentage of cells expressing CD25, statistically, significant differences were found with the rN-ANDV stimulus compared to the untreated cells from the rBCG-N-ANDV immunized group (*p* = 0.0447) (Figure 2F). The percentage of activation found with rBCG-N-ANDV was lower than the one reported in the rBCG-N-SARS-CoV-2 immunized group.

**Figure 3:**
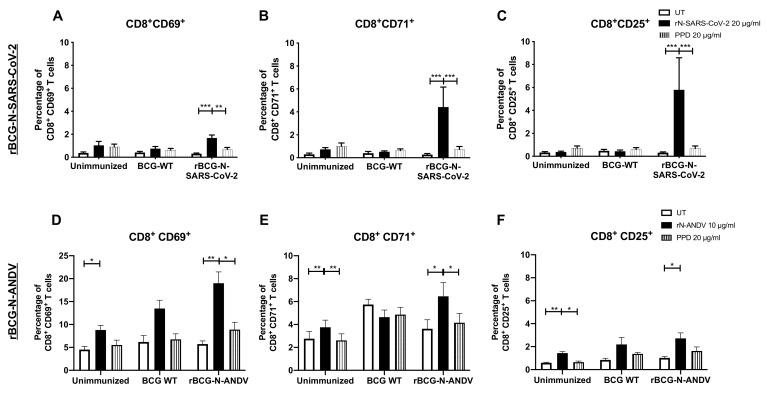
Determination of CD8^+^ T cell activation ex vivo after immunization. The expression of three different activation markers, CD69, CD71, and CD25, was evaluated ex vivo in cells from mice immunized with the recombinant BCGs. For rBCG-N-SARS-CoV-2, the activation of CD69 (**A**), CD71 (**B**), and CD25 (**C**) were evaluated in CD8^+^ T cells. For rBCG-N-ANDV, the activation of CD69 (**D**), CD71 (**E**), and CD25 (**F**) were evaluated in CD8^+^ T cells. Data are representative of 3 independent experiments. Each experiment considered a total of three mice per group. Statistical differences were assessed by two-way ANOVA with a *post hoc* Tukey test. *: *p* < 0.05; **: *p* < 0.01; ***: *p* < 0.001.

The CD69, CD71, and CD25 activation markers were also evaluated in CD8^+^ T cells (Figure 3). For rBCG-N-SARS-CoV-2, we detected a statistically significant increase in the expression of the CD69, CD71, and CD25 markers after rN-SARS-CoV-2 stimulation as compared to untreated or PPD-stimulated cells (*p* < 0.0001 and *p* = 0.0018 for CD69; *p* < 0.0001 and *p* = 0.0002 for CD71; and *p* = 0.0003 and *p* = 0.0007 for CD25; respectively for UT and PPD) (Figure 3A–C). The activation levels found for CD8^+^ T cells were lower than those seen for CD4^+^ T cells in Figure 2. A similar response was found for the cells from rBCG-N-ANDV. A statistically significant increase in the expression of the CD69, CD71, and CD25 markers was detected when the cells were stimulated with the rN-ANDV protein compared to untreated or PPD-stimulated cells (*p* = 0.0021 and *p* = 0.0197 for CD69; *p* = 0.0130 and *p* = 0.0319 for CD71; and *p* = 0.0247 for CD25; respectively for UT and PPD) (Figure 3D,E). The proportion of activated CD8^+^ T cells was similar to that reported for CD4^+^ T cells from the rBCG-N-ANDV immunization experiment. Finally, an increase in the activation of CD69 (*p* = 0.0379) and CD25 (*p* = 0.0020) was observed in the unimmunized mice in the presence of the rN-ANDV protein, suggesting a possible basal level of immunogenicity for this antigen.

### 3.4. Immunization with the Recombinant BCGs Promotes Antiviral Cytokine Secretion in T Cells Stimulated Ex Vivo

**Figure 4:**
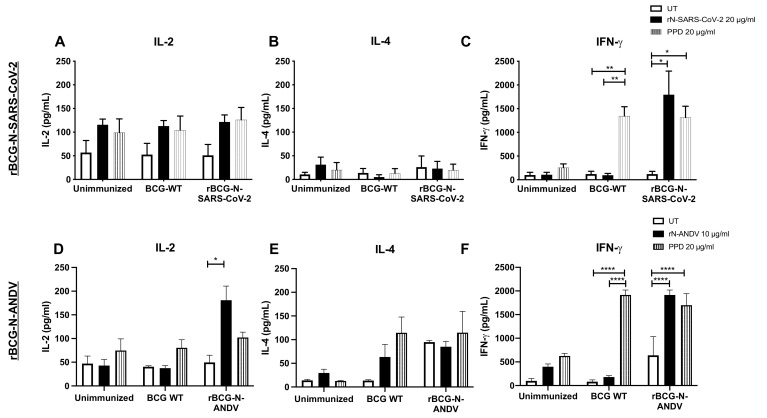
Determination of cytokines secreted ex vivo in immunized mice. The determination of IL-2, IL-4, and IFN-γ were evaluated ex vivo from mice immunized with both recombinant BCGs. For rBCG-N-SARS-CoV-2, the secretion of IL-2 (**A**), IL-4 (**B**), and IFN-γ (**C**) were evaluated in CD8^+^ T cells. For rBCG-N-ANDV, the activation of IL-2 (**D**), IL-4 (**E**), and IFN-γ (**F**) were evaluated in CD4^+^ T cells. Data are representative of 3 independent experiments. Each experiment considered a total of three mice per group. Statistical differences were assessed by two-way ANOVA with a *post hoc* Tukey test. *: *p* < 0.05; **: *p* < 0.01; ****: *p* < 0.0001.

To address the cytokine secretion profile induced by immunization, the concentration of different cytokines was evaluated in the supernatants of T cells stimulated ex vivo with antigens (Figure 4). As a parameter of activation of T cells, IL-2 was evaluated, which showed a higher, but not statistically significant, secretion when cells were stimulated with rN-SARS-CoV-2 or PPD compared to the untreated cells (Figure 4A). Low concentration values were found for IL-4 in all groups with the different stimuli evaluated (Figure 4B). The concentration of IFN-γ was significantly higher in the cells obtained from rBCG-N-SARS-CoV-2-vaccinated mice when stimulated with either rN-SARS-CoV-2 (*p* = 0.0332) or PPD (*p* = 0.0108), as compared to unstimulated cells (Figure 4C). We also detected a significant increase in IFN-γ in response to PPD stimulation in cells obtained from BCG-WT-immunized mice compared to untreated (*p* = 0.0059) and rN-SARS-CoV-2 (*p* = 0.0029) stimulated cells.

The concentrations of cytokines detected in the samples from rBCG-N-ANDV were similar to those reported for the rBCG-N-SARS-CoV-2 vaccine prototype. We observed a significant increase in IL-2 concentration in the presence of rN-ANDV in the mice immunized with the rBCG-N-ANDV compared to untreated cells (*p* = 0.0464) (Figure 4D). No significant differences were reported for the IL-4 concentrations in all the evaluated groups (Figure 4E). We found an increase in IFN-γ concentrations in the cells from the mice immunized with the BCG-WT stimulated with PPD compared to the untreated and the rN-ANDV stimulated cells (*p* < 0.0001) (Figure 4F). Stimulating the cells from rBCG-N-ANDV immunized mice with PPD (*p* < 0.0001) or the rN-ANDV protein (*p* < 0.0001) increased the concentration of IFN-γ compared to the untreated cells (Figure 4F).

### 3.5. Immunization with Recombinant BCG Vaccine Promotes the Induction of Trained Immunity Parameters

**Figure 5:**
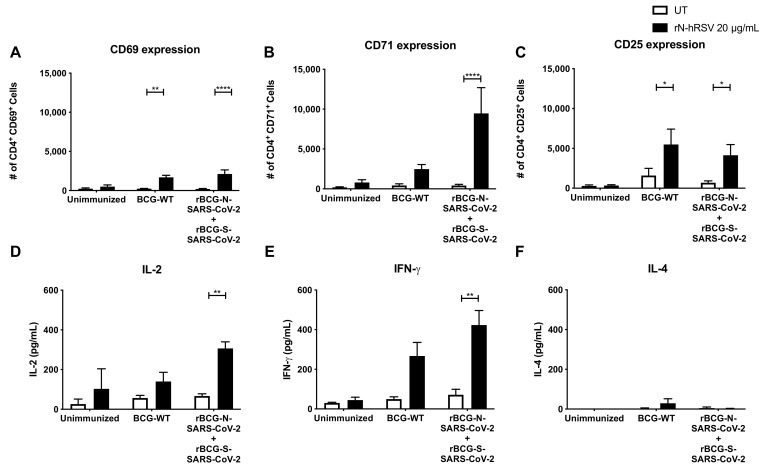
Evaluation of CD4^+^ T cell activation and cytokines concentration secreted ex vivo upon stimulation of purified T cells with a non-related antigen. The expression of the activation markers CD69 (**A**), CD71 (**B**), and CD25 (**C**) was evaluated by flow cytometry in purified T cells upon stimulation with the N-hRSV protein, an unrelated antigen. These purified T cells were obtained from mice unimmunized, immunized with BCG-WT, or immunized with a mix of rBCGs expressing either the N- or the S-SARS-CoV-2 protein. Accordingly, the secretion of IL-2 (**D**), IL-4 (**E**), and IFN-γ (**F**) were evaluated in the supernatants obtained from the stimulation of these T cells. Data are representative of 2 independent experiments. Each experiment considered a total of three mice per group. Statistical differences were assessed by two-way ANOVA with a *post hoc* Bonferroni test. *: *p* < 0.05; **: *p* < 0.01; ****: *p* < 0.0001.

As BCG has been reported to induce trained immunity in mice and humans, we evaluated different parameters related to this response for our recombinant BCGs. We evaluated the activation of T cells and the secretion of cytokines elicited by an experimental setup following the same conditions as the one described for the rBCG-N-SARS-CoV-2 (a single dose with 1 × 10^5^ CFU and an ex vivo co-culture stimulation assay) but immunizing instead with a mix of two recombinant BCGs, one being the rBCG-N-SARS-CoV-2 and another one expressing the S-protein of the same virus (rBCG-S-SARS-CoV-2). The activation of T cells and the secretion of cytokines were evaluated upon stimulation of purified T cells with an unrelated antigen, i.e., the hRSV Nucleoprotein (N-hRSV). We detected a statistically significant increase in the expression of CD69 and CD25 by CD4^+^ T cells upon stimulation with the N-hRSV protein in the mice immunized with the BCG-WT and the mix of recombinant BCGs (*p* = 0.0017 and *p* < 0.0001 for CD69; *p* = 0.0303 and *p* = 0.0491 for CD25, respectively) (Figure 5A–C). This was also seen for CD71 only for the mice immunized with the mix of rBCGs (*p* = 0.0002). We also detected a statistically significant increase in the secretion of pro-inflammatory cytokines IL-2 (*p* = 0.0261) and IFN-γ (*p* = 0.0078) upon stimulation with the N-hRSV protein in the mice immunized with the mix of recombinant BCGs (Figure 5D,F). A statistically significant increase was also seen for the mice immunized with BCG-WT for IFN-γ (*p* = 0.0287). No differences were seen in the secretion of IL-4 (Figure 5E).

The secretion of IL-6 and TNF-α, which are cytokines previously related to the trained immunity response, was evaluated in the supernatants of the ex vivo assays performed for the rBCG-N-SARS-CoV-2 vaccine alone (Supplemental Figure S2). Higher levels of IL-6 were detected in the groups immunized with either BCG-WT or rBCG-N-SARS-CoV-2 (Supplemental Figure S2A). This increase was seen when the cells were stimulated with either the N-SARS-CoV-2 protein or PPD. No IL-6 was detected when the cells were not stimulated. Remarkably, basal levels of IL-6 were detected in unimmunized mice when treated with the stimulus, but the concentrations detected were lower than those reported for immunized groups. No differences were seen in TNF-α concentrations when the cells from all the experimental groups were stimulated with the N-SARS-CoV-2 protein (Supplemental Figure S2B). However, stimulation with PPD resulted in increased concentrations of TNF-α when evaluated in all the experimental groups. Taken together, these results suggest that BCG promotes a trained immunity profile in the immunized mice, although further assays are required to elucidate this fully.

### 3.6. The Secretion of Specific Antibodies Depends on the Dose and the Number of Immunizations Administrated

**Figure 6:**
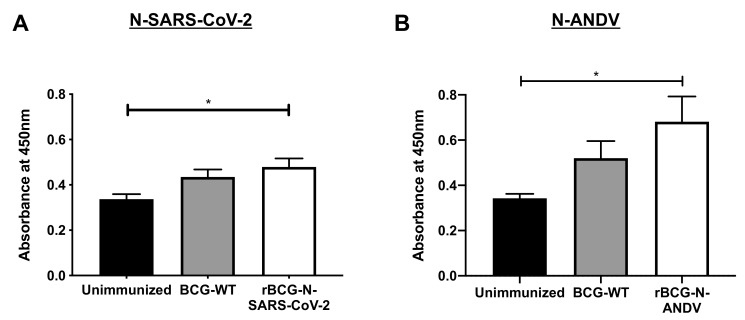
Determination of specific antibodies induced by immunization with the recombinant BCGs. ELISAs were performed to measure total antibodies against the nucleoproteins in samples collected on day 24 post-immunization (rBCG-N-SARS-CoV-2) (**A**) or 28 post-immunization (rBCG-N-ANDV) (**B**). Data are representative of 3 independent experiments. Each experiment considered a total of three mice per group. Statistical differences were assessed by Kruskal–Wallis test with a *post hoc* Dunn’s test. *: *p* < 0.05.

The humoral immune response was evaluated for both recombinant vaccines. Because each vaccination was performed using different doses and experimental schemes, we evaluated the response using blood samples collected upon euthanasia (Figure 6). Low levels of anti-N-SARS-CoV-2 antibodies were detected overall after immunization with rBCG-N-SARS-CoV-2. Although modest, a statistically significant increase in the absorbance value for the sera obtained from the rBCG-N-SARS-CoV-2-immunized mice could be detected compared to the unimmunized mice (*p* = 0.0241) (Figure 6A). Similar results were found when the anti-N-ANDV antibodies were measured. There was a modest but statistically significant increase in the levels of antibodies against the N-ANDV protein, as determined by measured absorbance values for the sera obtained from mice immunized with the rBCG-N-ANDV vaccine compared to the unimmunized mice (*p* = 0.0184) (Figure 6B). This suggests that as the maximum threshold value for these assays in our settings is 2.0, and the data obtained for these vaccines are close to 0.6, there is not marked increase in the humoral responses upon immunization with these recombinant BCG prototypes. However, the antigen-specific OD values for these antibodies did increase after immunization. The administration of booster doses with recombinant proteins or a challenge with the respective viruses may induce a more potent humoral immune response, although further analyses are required to confirm this [46].

## 4. Discussion

*Mycobacterium bovis* Bacillus Calmette-Guérin (BCG) is an attenuated strain of *M. bovis*, obtained after about 230 in vitro passages, and the only current vaccine for the prevention and treatment of the infection caused by *M. tb* [1,2,3]. This live-attenuated vaccine has been widely used for over a century to prevent tuberculosis disease, and it is one of the most used vaccines worldwide, with a well-characterized and documented safety profile [4,5]. Remarkably, BCG has also been used as a vector for the heterologous expression of recombinant antigens, with a positive correlation of protection against diseases caused by measles virus, hepatitis B virus, respiratory syncytial virus, and *Bordetella pertussis*, among others [5,45,46,48,49,50,51]. Hence, the rationale for using BCG as a vector for the expression of heterologous proteins, inducing a well-balanced immune response, leaning towards a Th1 polarization upon viral infections.

Here, we report data regarding two recombinant BCGs expressing either the N-SARS-CoV-2 or the N-ANDV proteins and their capacity to induce adaptive and trained immunity traits in mice. As reported for other rBCG vaccines, we observed favorable safety profiles for both vaccine prototypes [5,45,46,48,49,50,51]. Notably, mice immunized with the rBCG-N-SARS-CoV-2 vaccine in a single, low dose of this prototype exhibited no differences in weight changes, clinical score, and behavior compared to unimmunized or BCG-WT immunized mice. Accordingly, administration of two doses of 10^8^ CFU of rBCG-N-ANDV was well tolerated by mice, without significant weight change, clinical score, behavior, or posture parameters associated with systemic adverse effects compared to those that might be associated with systemic adverse effects in BCG-WT-immunized or unimmunized mice. Likewise, local reactions for the rBCG-N-ANDV were similar to those of BCG-WT or other rBCGs immunization [48]. These results indicate that the administration of these recombinants prototypes in mice exhibits a good safety profile and suggests the possibility of escalation of these vaccines onto clinical assays, as performed with other rBCGs, such as those expressing hRSV antigens [48,58].

Recombinant BCG vaccines have shown to be inducers of Th1 immune response, eliciting long-term, antigen-specific CD4^+^ and CD8^+^ T cell priming, associated with effective antiviral immunity [46,48,49,50,51,58]. Here, the immunogenicity of a single, low dose of the vaccine prototype against SARS-CoV-2 was also evaluated. It has been shown that rBCGs are safe vaccines that induce strong CD4^+^ and CD8^+^ T cell activation with polarization towards a Th1 CD4^+^ T cells profile, capable of secreting high amounts of IFN-γ [48]. In addition, several reports show that the SARS-CoV-2 Nucleoprotein is a good target for vaccine development as it is conserved and stable, presents fewer mutations over time, is abundant during infection, and is highly immunogenic [59], characteristics that could explain the statistically significant increase in the percentage of activated CD4^+^ and CD8^+^ T cells when stimulated with the purified N-SARS-CoV-2 protein at a higher concentration. In this work, we observed that immunization with rBCG-N-SARS-CoV-2 caused a significant increase in the frequency of CD4^+^ and CD8^+^ T cell activation markers (except for the CD69 marker in the CD4^+^ T cell subset), which suggests a positive contribution to the host immune response against SARS-CoV-2, and proposes that this formulation could be a potential candidate for escalation into clinical testing [60].

Several reports have shown that cellular immunity, particularly CD8^+^ T cells, is vital for generating protective and cross-protective responses to ANDV infections [61,62,63,64]. Regarding HCPS, protection in the absence of neutralizing antibodies has been reported after immunization with adenoviral vectors encoding for different viral antigens [62]. An ANDV DNA vaccine against four different species of this virus protected against infection by this virus in Syrian hamsters, despite eliciting low neutralizing antibody titers [63]. Here, vaccination with the rBCG-N-ANDV vaccine generated antigen-specific activation of CD4^+^ and CD8^+^ T cells, evidenced by an increased percentage of T cells expressing the activation markers CD69, CD71, and CD25, after ex vivo stimulation with the recombinant N-ANDV antigen, compared to unimmunized animals.

To date, there are no approved vaccines against SARS-CoV-2 that use the N protein of this virus in a recombinant vector such as BCG. However, DNA vaccine formulations expressing the N protein of SARS-CoV are being tested, showing a strong induction of humoral and cellular immune responses [65,66]. Our vaccine candidate showed significant production of IL-2 and IFN-γ, two cytokines of the cellular responses specific against SARS-CoV-2 [67], and cytokines that are also related to a Th1 phenotype. Consistently, we did not observe elevated amounts of IL-4, a cytokine typically secreted by cells with a Th2 phenotype [68]. This suggests that our vaccine induces suitable responses against SARS-CoV-2 by promoting a Th1-polarized phenotype and low Th2-biased responses, thus reducing the potential for enhanced respiratory disease (ERD) [69,70]. Accordingly, different reports with ANDV disease models have shown protection from infection or disease upon vaccination with recombinant nucleoproteins from these viruses [61,62]. The rBCG-N-ANDV vaccine was generated to prevent severe HCPS. Although the molecular mechanisms underlying HCPS are not completely clear yet, data from clinical samples and animal models indicate solid and dysbalanced Th1/Th2 immune responses participate in this response, increasing dysregulation of airway epithelial cells, leading to pulmonary oedema and heart failure [45,48,49]. The generation of an antigen-specific cellular response was also associated with increased IFN-γ secretion after N-ANDV stimulation of splenocytes from rBCG-N-ANDV immunized mice compared to unimmunized. The secretion of IL-4 was also increased in the immunized group compared to the unimmunized group; however, IFN-γ levels were higher.

Antibodies are critical players in the protection against infectious diseases. Mainly, neutralizing antibodies play a critical role during viral infections [71,72,73,74]. For coronaviruses, most antibodies are directed to the S and N antigens of the virus during natural infections [75]. We found that the rBCG-N-SARS-CoV-2 vaccine induced high titers of total IgG specific against the Nucleoprotein of this virus. Accordingly, a mild but significant increase in serum N-ANDV specific IgG levels was observed in rBCG-N-ANDV-immunized mice compared to unimmunized mice, suggesting that a specific humoral response occurs after vaccination. Despite not performing a challenge for either of the immunization schedules, the mild increase seen in our models maybe be related to higher IFN-γ secretions induced by the immunization. This increase in IFN-γ concentrations may favor the proliferation and differentiation of B cells into effector plasma cells, which might trigger antibody secretion, specifically of the IgG2a isotype in mice [46,76]. It remains to be determined if rBCG vaccination induces a suitable humoral immune response that can protect the host from COVID-19 or lethal HCPS in mouse models. In this line, the role of neutralizing antibodies, the induction of antibody-dependent cellular cytotoxicity, or cytotoxic-dependent cytotoxicity may be correlated with protection [77,78,79].

Remarkably, immunization with BCG has been shown to promote a trained immunity profile [80,81]. In this context, administration of BCG-WT elicited an increase in the activation of antigen-specific T cells and the secretion of cytokines against the N-ANDV antigen. This increase could be induced by the non-specific protective effects described as a consequence of BCG vaccination [80,81]. Similarly, we found that BCG-WT immunization promoted IgG secretion at concentrations higher than that observed in unimmunized mice but lower than those in rBCG-immunized mice. This increase could be associated with the ability of BCG to promote a Th1 polarization of T cells, which aids the proliferation and differentiation of B cells into antibody-secreting plasma cells [46,82]. In addition, increased expression of activation markers by CD4^+^ T cells and concentration of IL-2 and IFN-γ were detected in mice immunized with a mix of rBCGs expressing either the N-SARS-CoV-2 or the S-SARS-CoV-2 proteins when these cells were stimulated with the Nucleoprotein of hRSV. Previous reports have shown that immunization with BCG-WT reduced hRSV- and hMPV-related diseases parameters, so an increase in the activation of T cells and the secretion of these cytokines could explain this phenomenon [52,55,57]. In addition, increased secretion of IL-6 was detected when mice were immunized with either BCG-WT or rBCG-N-SARS-CoV-2, which is in line with the notion of trained immunity in those mice. However, further experiments are required to better understand the mechanisms underlying the unspecific immune responses elicited by BCG.

Taken together, the data presented herein suggest that the N-SARS-CoV-2 and the N-ANDV proteins are promising antigens for vaccine development using BCG as an antigen expression vector.

## Data Availability

All analyzed and raw data are available upon reasonable request to the corresponding author through email after the publication of this article. A signed data access agreement will be requested to share the data.

## Supplementary Materials

The following supporting information can be downloaded at https://www.mdpi.com/article/10.3390/vaccines10050721/s1: Figure S1: Evaluation of the expression of the recombinant protein on the rBCGs by Western Blot, Figure S2: Evaluation of cytokine concentrations secreted ex vivo related to trained immunity.
