# Supplementary material for: BCG-Based Vaccines Elicit Antigen-Specific Adaptive and Trained Immunity against SARS-CoV-2 and Andes orthohantavirus
Source: Vaccines (Basel). 2022 May 4;10(5):721. doi: 10.3390/vaccines10050721 (PMC9143576; doi:10.3390/vaccines10050721)
Supplement: Supplementary file 1 [file vaccines-10-00721-s001.zip › vaccines-1698077-supplementary.pdf]

Supplementary Information

BCG-Based Vaccines Elicit Antigen-Specific Adaptive and Trained Immunity against SARS-CoV-2 and *Andes orthohantavirus*

1. Materials and Methods

1.1 Generation of recombinant *Mycobacterium bovis* BCG strains expressing the Nucleoprotein of SARS-CoV-2 or ANDV.

To generate an rBCG expressing the Nucleoprotein of SARS-CoV-2 (N-SARS-CoV-2), the sequence of this gene was synthesized by GenScript using the reference sequence from the Wuhan-Hu-1 isolate (GenBank accession NC_045512.2). Then, the DNA coding sequence of the N-SARS-CoV-2 protein was cloned in the pMV361 plasmid through restriction enzymes. To generate an rBCG strain expressing the Nucleoprotein of ANDV (N-ANDV), we defined an *in-silico* cloning strategy. First, the *n-andv* gene was amplified by PCR and cloned into a TOPO-HisMAX pCDNA4 vector. Next, the *n-andv* gene was obtained from an ANDV sequence from the CHI-7913 isolate (GenBank accession AY228237.1). Later, the *n-andv* gene was amplified using the Phusion High Fidelity DNA polymerase (ThermoFisher #F530) and cloned into a pMV361 using the Gibson Assembly Cloning Kit (New England Biolabs #E5510). The presence of the genes in both the pMV361-N-SARS-CoV-2 and pMV361-N-ANDV constructions was confirmed by PCR and Sanger sequencing. Finally, the pMV361-N-SARS-CoV-2 and pMV361-N-ANDV plasmids were used to transform a wild-type BCG strain Danish 1331 (BCG-WT) through electroporation. The bacteria were allowed to recover for 24 h in a 7H9 medium at 37ºC and 150 RPM of agitation. Then, 200µL of these cultures were seeded onto 7H10 agar plates (Sigma-Aldrich, M0303), supplemented with 10% OADC (Sigma-Aldrich, M0678), and Kanamycin [20 µg/mL] (Sigma-Aldrich, 60615). Finally, the kanamycin-resistant rBCG colonies were grown on a 7H9 liquid medium (Sigma-Aldrich, M0178), supplemented with 10% OADC (Sigma-Aldrich, M0678), 0.05% Tween 80, and Kanamycin [20 µg/mL].

1.2 Expansion and characterization of rBCG strains.

The rBCG-N-SARS-CoV-2 and the rBCG-N-ANDV colonies were grown on a 7H9 liquid medium (Sigma-Aldrich, M0178), supplemented with 10% OADC (Sigma-Aldrich, M0678), 0.05% Tween 80 (Winkler), Fungizone 250 µg/mL (Thermo) and Kanamycin [20 µg/mL] for three weeks or until reaching an optical density (O.D. 600nm) between 0.8 to 1. The bacterial cultures were centrifuged and washed three times with 1X PBS - 0.05% Tween 80 to remove traces of OADC and stored at -80ºC in sterile PBS – 20% glycerol until use in a final stock of $4x10^8$ CFU/vial or $4x10^5$ CFU/vial. PCR and sequencing confirmation of both recombinant BCG strains were performed. The primers used to corroborate the presence of the genes by PCR were the following: *n-sars-cov2* gene Fwd 5′ – ATGTTTTTGTTTTTTCTTGTTTTTTTATTG – 3′ and Rev 5′- TTAAGTGGCCATTTATATATACT - 3′ primers. For *n-andv* were Fwd 5′ – GCCAAGACAATTGCGATGAGCACCCTCCAAGAATTGC – 3′ and Rev 5′-GTCGATCGTACGCTAGCTACAACTTAAGTGGCTCTTGGTTG - 3′ primers. External primers for pMV361 were Fwd. 5′ – CGGTGAGTCGTAGGTCGGGA – 3' and Rev 5' – GAGCAAGACGTTTCCCGTTG – 3'. For BCG 16s rRNA, the primers used were Fwd 5′ – ACG GTG GGT ACT AGG TGT GGG TTT C – 3' and Rev 5′- GAG TTT TAG CCT TGC GGC CGT- 3′.

1.3 Protein expression evaluation through Western Blot.

We performed Western blot assays to characterize the expression of the N-SARS-CoV-2 and N-ANDV by the recombinant BCGs. Before freezing the rBCG stock vials, 10 mL of each saturated culture were centrifuged and washed three times with 1X PBS- 0.05% Tween 80 and one time with 1X PBS. This pellet

was resuspended in extraction protein buffer (50 mM Tris, 0.6% SDS, 5 mM EDTA, and 1X cOmplete (Roche, 11697498001) protease inhibitor cocktail) and sonicated with five pulses of 30 seconds at an intensity of 15X (Microson ultrasonic cells disruptor XL), with 30 seconds of rest between each pulse. Total proteins extracted were quantified through Pierce BCA assays (Thermo - CAT#23225). 20 μg of total proteins were loaded into SDS-PAGE gels and run for 30 min at 90V and then for 90 min at 120V. Then SDS-PAGE was transferred into a nitrocellulose membrane using a Semi-Dry Power Blotter Transfer System (Thermo). Upon transfer, the membrane was blocked with 1X PBS - 10% BSA (Winkler) for two hours at room temperature. To confirm the expression of the N-SARS-CoV-2 and N-ANDV, we incubated with a mouse monoclonal anti-N antibody for SARS-CoV-2 diluted 1:500 (Sinobiological, cat 40143-MM05) or a monoclonal anti-N-ANDV mouse IgG1 antibody diluted 1:1,000 (Austral Biologicals, cat HNM-6021DZ1-5). Both antibodies were incubated O.N at 4ºC. After primary antibody incubation, the membranes were washed three times with 1XPBS- 0.05% Tween 20 solution, and then a Goat anti-mouse-HRP antibody (ThermoFisher) was incubated in a 1:2,000 dilution at room temperature for one hour. The membranes were washed three times, and a chemiluminescence reaction was performed with an ECL-Kit (Thermo). The membrane was visualized in a live view MyECL Imager (Thermo, cat 32106).

1.4 Bone marrow-derived Dendritic cells (BMDCs) culture.

Dendritic cells were differentiated from bone marrow precursors and then used as antigen-presenting cells for T cells *ex vivo* recall stimulation. The tibia and femur of 6- to 8-week-old BALB/c mice were collected after euthanasia. These bones were perfused with RPMI 1640 medium (GIBCO) to obtain the cells of the bone marrow. Erythrocytes were lysed with ACK solution for 5 min at RT and centrifuged at 400 x g for 5 min at 4ºC. Finally, $1.5x10^6$ cells/well were seeded into 24 well culture plates with RPMI 1640 complete medium (RPMI media, 10% FBS, 10 mM HEPES, 2 mM L-glutamine, 1mM Sodium pyruvate, 50 μM 2-mercaptoethanol, non-essential amino acids, penicillin [100 UI/mL], streptomycin [100 μg/mL], and [0.25 μg/mL] amphotericin B [0.25 μg/mL) supplemented with GM-CSF [1 μg/mL] for 5 days at 37ºC, 5%CO_2. Every 48 h, 500 μL of supernatants were removed, and 500 μL of fresh media were added. DC differentiation was routinely assessed by flow cytometry.

1.5 Flow Cytometry evaluation

Upon centrifugation, cells were washed with FACS buffer and stained with Fixable Viability Stain AF780 (BD Bioscience, Cat 565388) or AF700 (BD Bioscience, Cat 564997) diluted 1:1,000 for 20 minutes at 4ºC in the dark. After three washes, cells were incubated for 30 minutes at 4ºC in the dark with a mix of the following antibodies: anti-CD3 PerCP (BD Bioscience, Cat 561089), anti-CD4 BUV496 (BD Bioscience, Cat 612952), anti-CD8 BV510 (BD Bioscience, Cat 741051), anti-CD45 APC-Cy7 (BD Bioscience, Cat 561037), anti-CD69 BV786 (BD Bioscience, Cat 564683), anti-CD71 BUV395 (BD Bioscience, Cat 567254), and anti-CD25 BV421(BD Bioscience, Cat 566228) for the rBCG-N-ANDV assays or anti-TCR-β PerCP-Cy5.5 (BD Bioscience, Cat 560657), anti-CD4 BV650 (BD Bioscience, Cat 740446), anti-CD8 APC-Cy7(BD Bioscience, Cat 561967), anti-CD45 BV510 (BD Bioscience, Cat 567800), anti-CD69 BV786(BD Bioscience, Cat 564683), anti-CD71 BUV395 (BD Bioscience, Cat 567254), and anti-CD25 BV421(BD Bioscience, Cat 566228) for the rBCG-N-SARS-CoV-2 assays.

1.6 Quantification of cytokines secreted during co-cultures by ELISA.

To quantify the cytokines secreted after the co-culture of T cells and DCs, supernatants were collected and used directly (undiluted) to detect IL-2 (BD OptEIA™ Mouse IL-2 ELISA Set, CAT#555148), IL-4 (BD OptEIA™ Mouse IL-4 ELISA Set, CAT#555232), and IFN-γ (BD OptEIA™ Mouse IFN-γ ELISA Set, CAT#555138) following the instructions of the manufacturer. Plates were revealed using 1 mg/ml of 3,3′,5,5′-tetramethylbenzidine (TMB, BD Biosciences) at RT, protected from the light, for 15 minutes. Then, the reaction was stopped by adding 50 μL of 2 N H_2SO_4. Plates were analyzed in an ELISA reader at 450 nm and 560 nm (Multiskan Ex, Thermo Labsystems).

1.7 Quantification of specific IgG against SARS-CoV-2 antigens by ELISA.

To quantify the antigen-specific circulating IgG antibodies in immunized mice, 96 well ELISA plates were coated O.N. at 4ºC with 100 ng of rN-SARS-CoV-2 protein or 400 ng of rN-ANDV in coating buffer ($NaHCO_3$-Na_2CO_3 0,1 M; pH=9.5). Plates were then blocked with 200 µL of 1X PBS - 10% low-fat milk for 5 h at RT. Then, plates were washed three times with 200 µL of 1X PBS - 0.05% Tween 20, and sera samples (1:50 diluted for SARS-CoV-2 and ANDV) were incubated O.N. at 4ºC. Sera samples were incubated O.N. at 4ºC in ELISA plates coated with 100µg of total protein extracts of BCG-WT and blocked with 1x PBS – 5% BSA to remove cross-reactive antibodies. The next day, plates were washed three times and incubated with HRP-Goat anti-mouse IgG (H+L) (Life Technologies, N. Meridian rd., Rockford, IL 61101, USA) diluted 1/2,000 for 1 h at RT. Finally, plates were washed three times with 200 µL of 1X PBS - 0.05% Tween 20 and once with 1X PBS. Plates were revealed with 1 mg/ml 3,3′,5,5′-tetramethylbenzidine (TMB, BD Biosciences) at RT, protected from the light, for 15 minutes. The reaction was stopped by adding 50 µL of 2 N H_2SO_4. Plates were analyzed in an ELISA reader at 450 nm and 560 nm (Multiskan Ex, Thermo Labsystems).

2. Supplementary Figures

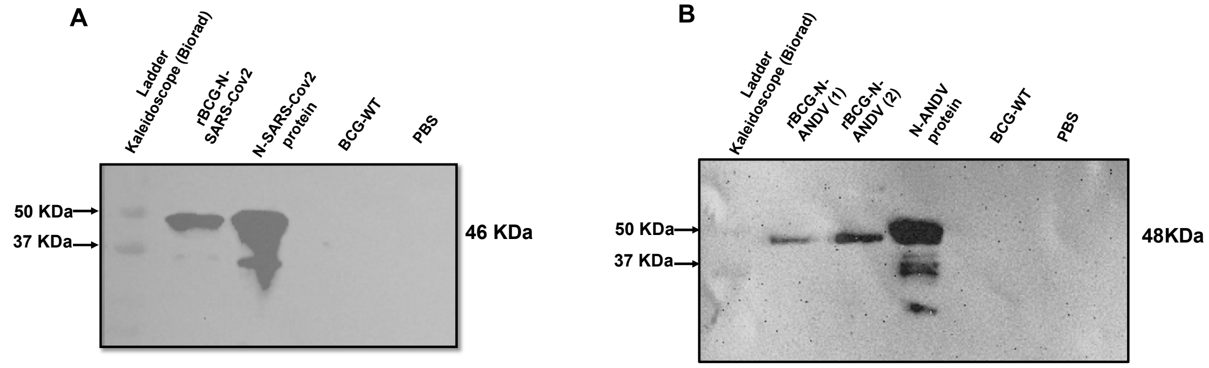

Figure S1. Evaluation of the expression of the recombinant protein on the rBCGs by Western Blot. The evaluation of the expression of the N-SARS-CoV-2 protein (**A**) or the N-ANDV protein (**B**) was evaluated for the recombinant BCGs expressing the corresponding protein through Western Blot assays.

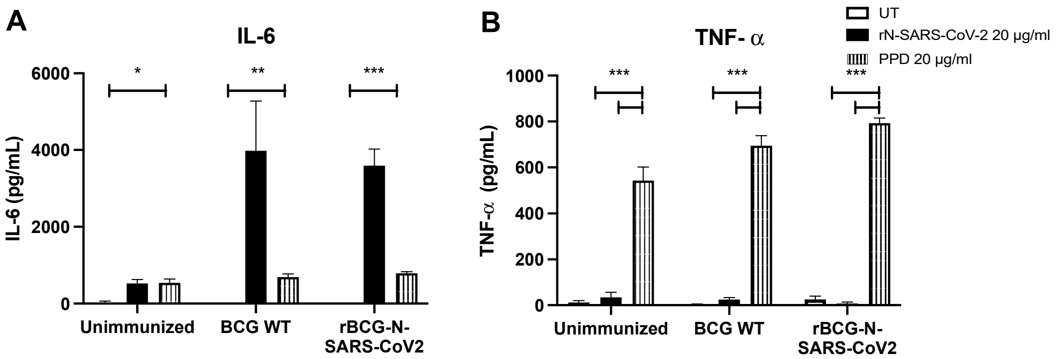

Figure S2. Evaluation of cytokine concentrations secreted ex vivo related to trained immunity. The secretion of IL-6 (**A**) and TNF-a (**B**) was evaluated ex vivo in immunized mice with the recombinant BCG for SARS-CoV-2. Data are representative of 3 independent experiments. Each experiment considered a total of three mice per group. Statistical differences were assessed by two-way ANOVA with a post hoc Tukey test. *: p<0.05; **: p<0.01; ***: p<0.001.
